# Microfluidic Fabrication of Oleosin-Coated Liposomes as Anticancer Drug Carriers with Enhanced Sustained Drug Release

**DOI:** 10.3390/ma17225550

**Published:** 2024-11-13

**Authors:** Yoseph Seo, Yeeun Woo, Byeolnim Oh, Daehyeon Yoo, Hyeok Ki Kwon, Chulhwan Park, Hyeon-Yeol Cho, Hyun Soo Kim, Taek Lee

**Affiliations:** 1 Department of Chemical Engineering, Kwangwoon University, 20 Kwangwoon-ro, Nowon-gu, Seoul 01897, Republic of Korea; akdldytpq12@kw.ac.kr (Y.S.); yenn0706@kw.ac.kr (Y.W.); lovydh@kw.ac.kr (D.Y.); david981228@gmail.com (H.K.K.); chpark@kw.ac.kr (C.P.); 2 Department of Electronic Engineering, Kwangwoon University, 20 Kwangwoon-ro, Nowon-gu, Seoul 01897, Republic of Korea; o_byeolnim@kw.ac.kr; 3 Department of Bio & Fermentation Convergence Technology, Kookmin University, 77 Jeongneung-ro, Seongbuk-gu, Seoul 02707, Republic of Korea; chohy@kookmin.ac.kr

**Keywords:** microfluidic device, liposome, oleosin, anticancer drug, sustained release

## Abstract

Microfluid-derived liposomes (M-Lipo) exhibit great potential as drug and functional substance carriers in pharmaceutical and food science. However, the low liposome membrane stability, attributed to the liquid core, limits their application range. Oleosin, a natural surfactant protein, can improve the stability of the lipid nanoparticle membrane against various environmental stresses, such as heat, drying, and pH change; in addition, it can enable sustained drug release. Here, we proposed the fabrication of oleosin-coated M-Lipo (OM-Lipo) through self-assembly on a microfluidic chip and the evaluation of its anticancer drug (carmustine) delivery efficiency. Nanoparticle characterization revealed that the oleosin coating slightly lowered the membrane potential of M-Lipo and greatly improved their dispersibility. Additionally, the in vitro drug release profile showed that the oleosin coating improved the sustained release of the hydrophobic drug from the phospholipid bilayer in body temperature. Our results suggest that OM-Lipo has sufficient potential in various fields, based on its easy production, excellent stability, and biocompatibility.

## 1. Introduction

Cancer is one of the leading causes of human mortality worldwide [1]. Advancements in medicine have improved anticancer treatment via surgery, radiotherapy, and chemotherapy [2]. Among these, drug-based chemotherapy plays an important role in cancer treatment, but its efficacy is limited by poor drug pharmacokinetics, biodistribution, and solubility, as well as unintended side effects related to weak targeting [3,4]. Various drug delivery systems (DDS) have been proposed to address these challenges and increase the efficacy of anticancer drugs [5]. Nanoscale DDS platforms can improve anticancer drug bioavailability by increasing its half-life, controlling its release to target organs, and lowering its immunogenicity [6,7]. Lipid nanoparticles (LNPs) can improve poor anticancer drug solubility and passive targeting against solid tumors through their enhanced permeability and retention effect (EPR) [8,9,10,11]. These advantages render LNPs an important DDS platform in anticancer drug development [12,13,14].

Among the various types of LNPs, microfluid-derived liposomes (M-Lipo) are synthesized through self-assembly in microfluidic channels without strong physicochemical reactions [15]. Normally, M-Lipo are formed by mixing two different characteristic solutions on the microfluidic channel, and they can be manufactured in various sizes depending on the total flow rate (TFR), flow rate ratio (FRR), and composition of the solutions [16,17,18]. In addition, M-Lipo (<100 nm diameter) can be synthesized in bulk in one procedure with high lipid concentrations through short mixing sections with various patterns (e.g., chaotic and baffle mixing) on the microfluidic chip [19,20]. However, since M-Lipo have a liquid core, problems have been raised regarding their low stability to environmental stress, particle aggregation during storage (especially under low temperature), and early drug burst release [21,22]. To solve these problems, DDS studies are being conducted to improve the membrane stability of M-Lipo through surface modification based on various materials [18,23,24].

Recent studies on LNP’s surface modification have suggested that coating it with membrane structural proteins (oleosin) from oleosomes, which are abundant in plant seeds, can improve membrane stability [25]. Oleosin has a hairpin structure consisting of a widely spread amphipathic head part and a hydrophobic stem part [26]. Due to its surfactant-like amphipathic structure, it can be located in the membrane of the oleosome, which has a micelle structure, through hydrogen bonding and a hydrophobic interaction without any chemical treatment, and it can act as a natural emulsifier [27]. In addition, according to Li et al., the oleosin coating reduced the fluidity of the LNP’s membrane, thereby improving membrane stability and suppressing drug leakage [28].

Therefore, the main goal of this study was to evaluate the potential of oleosin-coated M-Lipo (OM-Lipo) as lipid-based DDS platforms for anticancer drug delivery. This builds upon our previous research, which explored genetically modified oleosin as a drug delivery carrier using oleosomes, natural lipid nanoparticles extracted from plant oils [29]. While oleosomes consist of a phospholipid monolayer micelle structure, their synthesis required multiple rounds of ultrasonication on ice to achieve nano-scale particles, introducing variability due to operator handling and making bulk-scale production challenging. In contrast, this study focuses on liposome-based lipid nanoparticles (LNPs) and investigates oleosin-coated liposomes (OM-Lipo), produced via a microfluidic system, enabling their more consistent and scalable production.

**Figure 1:**
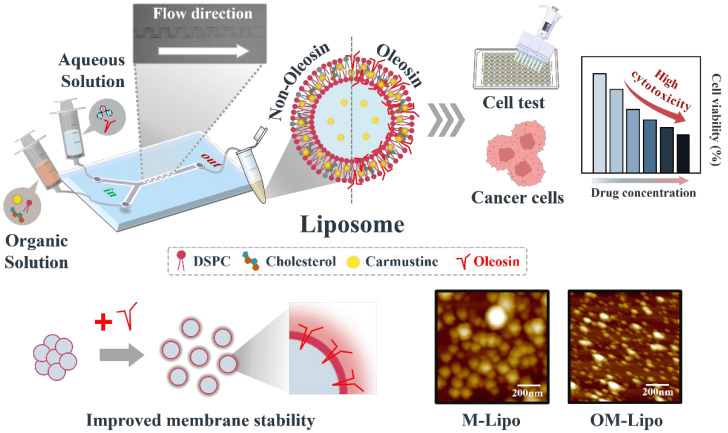
Schematic diagram of the fabrication of microfluid-derived liposome with oleosin coating.

This research proposes that OM-Lipo, with a size below 100 nm, can be produced through self-assembly on a microfluidic chip without the need for strong physical or chemical reactions, providing a stable and reproducible method for large-scale drug carrier production. We performed various tests to verify whether oleosin-based surface modification could affect M-Lipo functionality (Figure 1). First, a stability test based on nanoparticle characterization (dynamic light scattering, DLS, and zeta-potential measurement) was conducted to confirm whether the oleosin coating affects LNP stability. To verify the possibility of controlled drug release of M-Lipo using oleosin, a drug release profile was constructed based on reverse-phase high performance liquid chromatography (RP-HPLC). Finally, the cytotoxicity and anticancer drug delivery ability of OM-Lipo were evaluated through cell experiments with normal (L929) and breast cancer (SK-BR-3) cell lines.

## 2. Materials and Methods

### 2.1. Materials

Cholesterol, carmustine, and sodium hydrogen carbonate were purchased from Sigma-Aldrich (St. Louis, MO, USA). Moreover, 1,2-disteroylphosphatidylcholine (DSPC) was brought from Avanti Polar Lipids, Inc. (Alabaster, AL, USA). Methyl alcohol (99.5%), ethyl alcohol anhydrous (99.9%), acrylamide, and ammonium persulfate were obtained from Daejung Materials and Chemicals (Siheung-si, Gyeonggi-do, Republic of Korea). Hexane (96%) was purchased from Junsei Chemical Co. Ltd. (Tokyo, Japan), and *N*,*N*,*N*′,*N*′-tetramethylethylenediamine from Bio-Rad Laboratiories, Inc. (Hercules, CA, USA). Next, 1.5 M Tris-HCl buffer solution (pH 8.8) and 1 M Tris-HCl buffer solution (pH 6.8) were obtained from Biosesang (Seongnam-si, Gyeonggi-do, Republic of Korea). The 10× phosphate-buffered saline (PBS) (pH 7.4), 0.25% 1× Trypsin-EDTA, and 100× antibiotic-antimycotic were bought from Thermo Fisher Scientific (Waltham, MA, USA). Fetal bovine serum (FBS) was purchased from GW Vitek (Seoul, Republic of Korea). The distilled water (DIW) used in the experiment was obtained using the Milli-Q system (Burlington, MA, USA).

### 2.2. Oleosin Extraction

Oleosin was extracted from rapeseed (*Brassica napus* L., variety Alizze), purchased from the Korean market based on the method of Plankensteiner et al. [30]. Briefly, washed rapeseed was incubated in 0.1 M NaHCO_3_ (pH 9.5) at a seed-to-solution ratio of 1:7 (*w*/*w*) for 16 h at 4 °C. The mixture was homogenized for 2 min at 26,000 rpm using a blender (Shinil Electronics, Seoul, Republic of Korea), and the solid residues were removed by filtering the homogenate through a triple layer of cheesecloth. The filtered liquid was then centrifuged at 10,000× *g* for 30 min at 4 °C to collect the top cream layer, which contained the oleosomes. To further purify the cream, the same centrifugation process was performed sequentially with a new ratio of 1:4 (*w*/*w*) using 0.1 M NaHCO_3_ (pH 9.5) and then with DIW. Next, the washed cream layer was sequentially treated with three different organic solvents (methanol, hexane, and ethanol) at a ratio of 1:2 (*w*/*v*) to extract oleosin. After each organic solvent treatment, the mixture was incubated at 150 rpm for 10 min and centrifuged at 4700× *g* at room temperature. Each organic solvent-based washing step was repeated three times for each solvent. After the final ethanol wash, the remaining oleosin pellet was dispersed in 10 mL of DIW and treated in a bath-type ultrasonicator (40 kHz) at 25 °C for 10 min. The obtained oleosin pallet was dispersed in 10 mL of DIW using a bath-type ultrasonicator (40 kHz) at 25 °C for 10 min. Finally, the oleosin pellet, from which the organic solvent was removed, was lyophilized and stored at −20 °C for further analysis.

### 2.3. SDS-PAGE

The oleosin sample (1 mg) was dispersed in 1 mL of SDS solution (2% *w*/*w*) using a vortex mixer, followed by incubation at 75 °C for 10 min. The solution was mixed with 2× Laemmli sample buffer at a ratio of 1:1 (*v*/*v*) and incubated for 20 min. The prepared solution was loaded into the SDS-PAGE gel, and electrophoresis was performed under 100 V for 10 min in the stacking gel with running buffer. Size-based band separation was conducted in the running gel at 120 V for 1 h. The compositions of the SDS-PAGE gel are listed in Supplementary Table S1. The three-color protein ladder (Dynebio Inc., Seongnam-si, Gyeonggi-do, Republic of Korea) was used as the molecular weight marker for proteins. After electrophoresis, the SDS-PAGE gel was stained with InstantBlue Coomassie Protein Stain (Abcam, Cambrdige, UK) for 1 h.

### 2.4. Manufacturing of Microfluidic Chip for M-Lipo

The microfluidic chip was designed based on the model reported by Matsuura-Sawada et al. [19]. The microfluidic device for M-Lipo synthesis was designed with two inlets, one for the sample and one for the buffer solutions, and one outlet (Figure S1). The design image depicts a microfluidic channel, featuring 20 rectangular-shaped baffles along its length. The 200 µm-wide main channel changes into a long and narrow path, outlined by a series of uniform baffles. These baffles, sized 100 × 150 µm, are positioned at intervals of 300 µm along the channel. Each baffle is spaced uniformly along the channel, creating a serpentine path that forces the fluid to flow around them.

The LNP synthesis microfluidic device comprised a polydimethylsiloxane (PDMS, Sylgard 184, Dow Corning, Midland, MI, USA) microchannel on a 1 mm-thick borosilicate glass substrate using soft lithography techniques. First, the master mold for the device, with a 100 μm-high microstructure, was patterned with a SU-8 photoresist (SU-8 2050, Kayaku, Westborough, MA, USA) using a conventional photolithography process. The SU-8 master mold was coated with tridecafluoro-1,1,2,2-tetrahydrooctyl-1-trichlorosilane (Sigma-Aldrich, St. Louis, MO, USA) to facilitate PDMS replication. A PDMS device with a thickness of 5 mm was punched with two inlets and one outlet, each 0.75 mm in diameter, and then bonded to a glass substrate through oxygen plasma treatment (COVANCE, Femto Science, Hwaseong-si, Gyeonggi-do, Republic of Korea).

### 2.5. M-Lipo Preparation

M-Lipo were fabricated through a microfluidic chip incorporating a baffle mixing section, utilizing two solutions based on organic and aqueous solutions. Ethanol was employed as the solvent in the organic solution, which included two different molar ratios (mM) of DPSC/cholesterol (6:4 and 7:3, respectively). Carmustine, at a final concentration of 10 µg/mL, was dissolved in the organic solution for characterization. The aqueous solution was composed of 1× PBS. When producing M-Lipo with oleosin, the pH was adjusted to a slightly acidic condition (pH 4) to mitigate the aggregation of oleosin acting as a surfactant in the aqueous solution. During the injection of the respective solutions into the microfluidic chip, Microbore tubing (LK Lab Korea, Seoul, Republic of Korea) was attached to a syringe (Korea Vaccine, Ansan-si, Gyeonggi-do, Republic of Korea). Each syringe was then connected to the microfluidic chip channel. For M-Lipo synthesis, the TFR and FRR between the solutions were adjusted using a syringe pump (Chemyx Inc., Stafford, TX, USA). To facilitate the continuous heating of solutions, considering the characteristic transition temperature of DSPC at 55 °C, we used a carbon film heater with a sufficient size to cover the syringe. This heater was connected to an automatic temperature control device (Figure S2). The resulting M-Lipo were purified using a 14 kDa dialysis membrane (Repligen, Waltham, MA, USA) to remove impurities, such as ethanol, and subsequently stored at a refrigerated storage temperature (4 °C).

### 2.6. M-Lipo Characterization

To characterize the fabricated nanoparticles, the particle size (hydrodynamic diameter), polydispersity index (PDI), and zeta potential were measured using ELS-Z-200ZS (Otsuka, Osaka, Japan). The particle size and PDI were measured using DLS. The samples were measured at 25 °C (room temperature) without additional dilution, using a scattering angle of 165° and a laser wavelength of 663.2 nm. Zeta potential was determined via electrophoretic light scattering at a scattering angle of 15° at 25 °C. Before investigation, all samples were dialyzed for 2 h using a 14 kDa dialysis membrane under room temperature to prevent drug release. All measurements were conducted in triplicate.

Atomic force microscopy has been widely utilized in the detailed characterization of drug delivery systems, including liposomes, providing critical insights into their surface morphology, stability, and dispersity under various conditions [31,32]. In this study, atomic force microscopy (AFM; XE7, Park Systems, Suwon-si, Gyeonggi-do, Republic of Korea) imaging was used to characterize the surface and stability of M-Lipo depending on their oleosin coating. Briefly, 2 μL of liposome samples (3% formalin) were immobilized on a silicon wafer and allowed to dry. AFM images were obtained in non-contact mode. PPP-NCHR (Park Systems) was used as the cantilever, and silicon tips with a resonance frequency of 330 kHz and a spring constant of 42 N/m were selected at a scan rate 0.50 Hz and a resolution of 512 pixels. Surface roughness data were inherently included as part of the AFM imaging output. The obtained AFM images were processed using Park XEI software version 5.2.0 Build 1 (Park Systems).

### 2.7. Drug Encapsulation Efficiency (EE) and In Vitro Drug Release Profile

The EE (%) of carmustine in M-Lipo was determined using the following equation: EE(%) = (Total drug − Free drug)/Total drug × 100. Briefly, it was derived by subtracting the quantity of unencapsulated free drug remaining in the solution from the initial quantity of drug employed (total drug), followed by multiplication by 100%. The separation of the unloaded drug was carried out using a 10 kDa MWCO (Merck Millipore, Darmstadt, Germany) Amicon^®^ Ultra-Centrifugal Filter.

Drug release was quantified via RP-HPLC. Separated samples were filtered with 0.2 µm syringe filter (Advantec, DISMIC-13HP, Tokyo, Japan). The filtered sample was then analyzed through RP-HPLC (Agilent, 1260 Infinity II, Santa Clara, CA, USA) using a C18 column (5 µm, 4.6 × 250 mm, Youngjin Biochrom, Seongnam, Republic of Korea) at 50 °C column temperature. The injection volume used for analysis was set to 5 µL. The flow rate of the mobile phase was 0.5 mL/min, and the isocratic mobile phases used were acetonitrile (A) and water (B) at a 95/5% ratio. Carmustine was detected via UV spectroscopy at a 280 nm wavelength.

Carmustine is known to be unstable in the water phase (e.g., 1× PBS solution), and this was verified in our experiment, in which 30 µg/mL carmsutine were measured with RP-HPLC at 1-day intervals (Figure S3). Therefore, the in vitro drug release profiles were conducted based on coumarin-6, which is considered an alternative to carmustine [33]. The EE (%) of coumarin-6 in M-Lipo was determined as described above, using a Synergy LX microplate reader (BioTek, Winooski, VT, USA) with a fluorescence spectrophotometer. To analyze the drug release profiles of M-Lipo and OM-Lipo, a total of 10 mL coumarin-6-loaded liposome solution in 1 kDa dialysis membrane was incubated with 90 mL 1× PBS buffer (pH 7.4, containing 0.5% *w*/*v* SDS) under constant shaking at 100 rpm and 37 °C in darkness. Next, 100 µL of the release solution was taken at each interval, and the same volume of 1× PBS solution was filled in at the same time. The samples were analyzed by the microplate reader with the excitation wavelength at 457 nm and emission at 501 nm. All the above experiments were repeated in triplicate.

### 2.8. Cell Line and Cell Culture

In this study, M-Lipo cytotoxicity was assessed using L929 mouse lung fibroblasts, sourced from the European Collection of Cell Cultures. Additionally, the efficiency of carmustine delivery within the nanoparticles was assessed using SK-BR-3 breast cancer cells from the Korean Cell Line Bank (KCLB, Seoul, Republic of Korea). Each cell line was cultured in DMEM media supplemented with 10% FBS and 1% antibiotic-antimycotic solution (100×), incubated at 37 °C in a CO_2_ incubator (Vision Scientific, Daejeon, Republic of Korea).

### 2.9. Cytotoxicity Test

Cell viability and drug delivery efficiency were assessed using the Viability Assay Kit (CELLO MAX^TM^, Anyang-si, Gyeonggi-do, Republic of Korea) through the WST-1 assay. To evaluate the cytotoxicity of M-Lipo, L929 mouse fibroblast cells were subjected to various conditions, confirming the impact of both M-Lipo and OM-Lipo on cell viability. Initially, 100 µL of medium containing 30,000 cells/well was dispensed into a 96-well plate (Corning Inc., Corning, NY, USA). After a 24 h incubation period, various concentrations of carmustine (1 to 40 µg/mL) were applied to individual wells, along with four distinct formulations of M-Lipo featuring different combinations of drug and oleosin. Following a 24 h incubation at 37 °C in a 5% CO_2_ incubator, the medium was completely removed, and a mixture of medium and Viability Assay Kit in a 10:1 ratio was added to each well, totaling 110 µL. The reaction continued for an additional 2 h under the same CO_2_ incubator conditions. After gently shaking the plate for about 1 min, absorbance at 450 nm was measured using the microplate reader, allowing the assessment of M-Lipo and OM-Lipo cytotoxicity based on cell viability.

### 2.10. Drug Delivery Efficiency Test

To assess the efficacy of M-Lipo in delivering anticancer agents, breast cancer cells (SK-BR-3) were seeded at 30,000 cells/well in a 96-well plate with 100 µL of fresh culture medium (DMEM) and incubated at 37 °C with 5% CO_2_ for 24 h. Subsequently, each well was treated with 10 µL of M-Lipo or OM-Lipo containing carmustine, as well as negative and positive controls consisting of medium and carmustine alone, respectively. After 24 h of treatment, the remaining drug and M-Lipo, not absorbed by the cells, were removed. A mixture of medium and Viability Assay kit at a ratio of 10:1 was added to each well, totaling 110 µL, followed by an additional 2 h incubation in the CO_2_ incubator. The absorbance in each well was then measured at 450 nm using the microplate reader.

## 3. Results

### 3.1. SDS-PAGE Analysis for Extracted Oleosin

SDS-PAGE was performed to assess the quantity and quality of the extracted rapeseed oleosin. Since oleosin has distinct structural properties, SDS-PAGE was performed under denaturing conditions. The SDS-PAGE analysis of the extracted oleosin showed two major protein bands with molecular weight values of 18 and 20 kDa (Figure S4). Among them, the thickest band was observed at 18 kDa; this SDS-PAGE profile was almost the same as that reported by Plankensteiner et al. [30].

### 3.2. Optimization of OM-Lipo Synthesis Conditions

M-Lipo particle size is greatly affected by the transition temperature of phospholipids, TFR, and FRR. These parameters must be optimized to synthesize M-Lipo smaller than 100 nm, which are suitable for effective anticancer drug delivery via the EPR effect. The DSPC used in this study has a high transition temperature (approximately 55 °C) compared to other phospholipids. While its high transition temperature could give high stability to DSPC-based liposomes in the body, it caused lipid aggregation when organic solutions were mixed and diluted with a water solution in the microfluidic channel at room temperature. In addition, this phenomenon disturbed the formation of M-Lipo (Figure S5). Therefore, to sustain the temperature of the organic solution above the DSPC transition temperature, the syringe and microfluidic chip were heated using a carbon film-based temperature control device during M-Lipo synthesis (Figure S2). In the selection process of the two flow conditions, to synthesize uniform M-Lipo smaller than 100 nm, known for their high drug delivery efficiency, TFR and FRR were set to 0.5 mL and 1:9, respectively [19].

**Figure 2:**
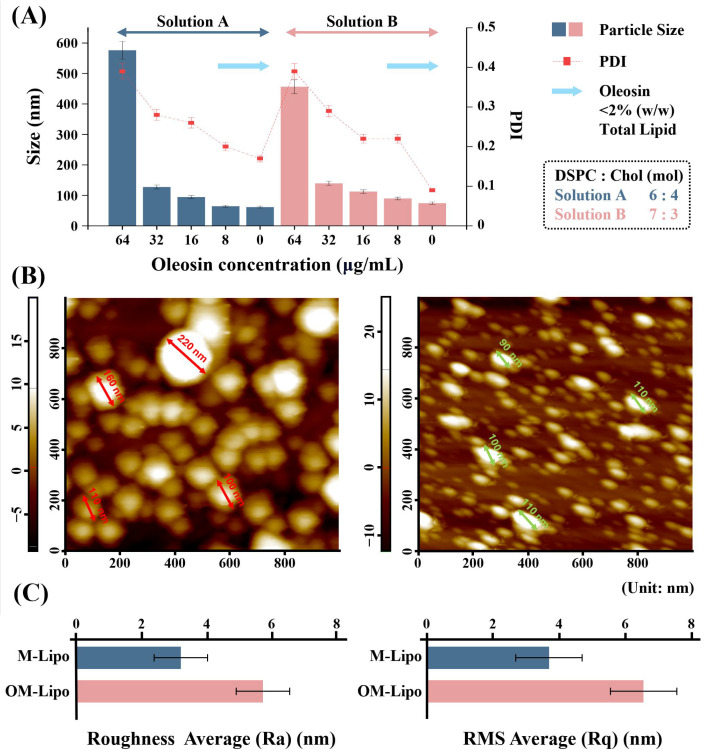
Particle characterization of fabricated microfluidic-derived liposomes. (**A**) Dynamic light scattering-based particle size and polydispersity index (PDI) analysis of OM-Lipo synthesized with varying oleosin concentrations (64, 32, 16, 8, and 0 µg/mL) and lipid formulations (DSPC ratios of 6:4 and 7:3). Bars represent particle size and squares indicate polydispersity index (PDI). OM-Lipo with a 6:4 ratio produced smaller particles compared to the 7:3 ratio. Oleosin concentrations below 2% (*w*/*w*) of the total lipid resulted in similar particle size and PDI to M-Lipo. (**B**) Atomic force microscopy-based liposome surface imaging analysis and (**C**) roughness measurements for M-Lipo and OM-Lipo.

OM-Lipo were synthesized by adding an oleosin water solution during M-Lipo manufacturing, based on optimized synthesis conditions (Figure 2A). To improve oleosin dispersibility, the water solution pH was adjusted to acidic condition (pH 3.5), and oleosin was then dispersed in the solution through sonication. The suitable oleosin concentration for membrane coating was selected by synthesizing OM-Lipo using various concentrations. According to particle characterization, overall, among the two DSPC/cholesterol molar ratios (6:4 and 7:3), 6:4 produced smaller-sized OM-Lipo than 7:3. Furthermore, while the addition of >2% (*w*/*w*) of oleosin compared to lipid increased OM-Lipo particle size to several hundreds of nanometers with high a PDI value, <2% (*w*/*w*) showed data almost similar to those of M-Lipo. As a result, to increase the delivery efficiency of anticancer drugs, we conducted subsequent experiments based on OM-Lipo using <2% (*w*/*w*) oleosin (i.e., 8 μg/mL), whose small size is most effective for generating the EPR effect.

### 3.3. Particle Stability Test

Particle aggregation increases particle size, and this phenomenon can be confirmed by observing changes in particle size based on particle characterization methods, such as AFM and DLS. The oleosin coating was performed using 2% (*w*/*w*) oleosin (8 μg/mL), which does not affect the particle size (Figure 2A). First, to determine the effect of the oleosin coating on liposomes’ dispersibility (about particle aggregation), each sample (M-Lipo and OM-Lipo) was fixed with formalin on bare silicon, and AFM-based surface analysis was performed. Figure 2B shows M-Lipo and OM-Lipo immobilized on bare silicon. Most M-Lipo particles were aggregated, sometimes into one, showing aggregates of over 200 nm. On the other hand, the OM-Lipo particles were evenly distributed, and fewer aggregates were observed compared to M-Lipo (Figure S6). Roughness analysis showed more high average roughness and root mean square roughness values in OM-Lipo (Figure 2C). AFM imaging analysis clearly showed that the addition of oleosin improved the dispersion of liposome solution.

**Figure 3:**
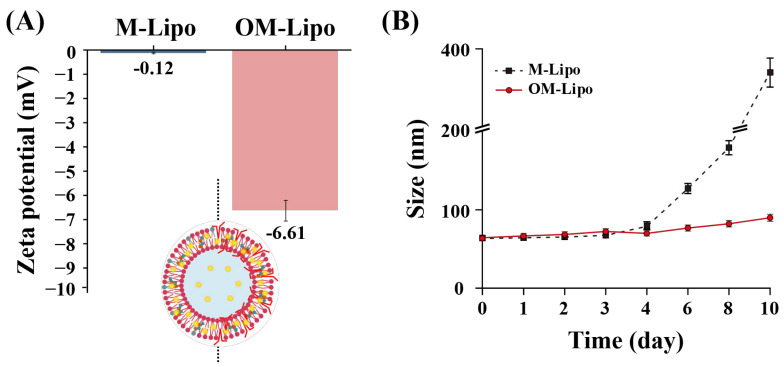
Particle stability test for M-Lipo and OM-Lipo in refrigerated storage (4 °C). (**A**) Initial zeta potential and (**B**) particle size measurement for 10 days.

Continuously, the effect of oleosin addition on the stability of the M-Lipo membrane was confirmed by observing the change in particle size during storage. Studies analyzing the interfacial properties of oleosin reported that the negative charge of oleosin can reduce the aggregation of coated particles (Figure 3A) [34]. In the zeta-potential measurement, the membrane potential of OM-Lipo (−6.61 mV) was lower than that of M-Lipo (−0.12 mV). This is attributed to the charge characteristics of oleosin, which has a strong negative charge at a neutral pH. As reported by Li et al., this result means that oleosin-based surface modification can prevent M-Lipo aggregation [28]. Stability tests during storage showed particle aggregation in M-Lipo after five days, but not in OM-Lipo (Figure 3B). Based on these results, we suggest the possibility that oleosin-based surface modification prevents LNPs’ aggregation.

### 3.4. In Vitro Release Kinetics

**Figure 4:**
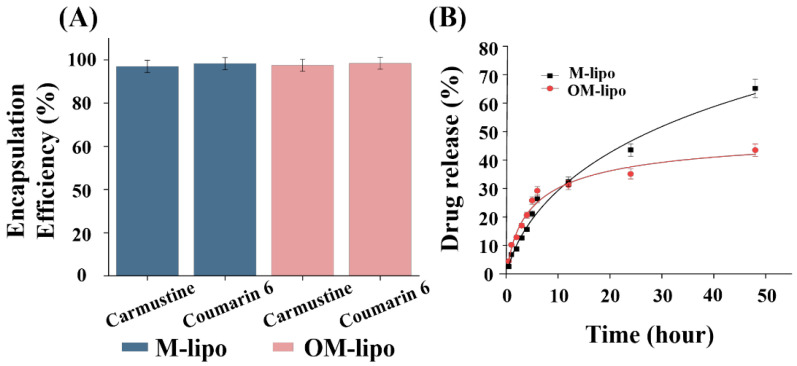
Evaluation of drug encapsulation efficiency and release profile of the fabricated carrier. (**A**) Drug encapsulation efficiency of carmustine and coumarin-6 in M-Lipo and OM-Lipo. (**B**) Coumarin-6-based cumulative drug release profile data.

To test whether oleosin could improve the sustained drug release of LNPs, we compared the drug release profiles of M-Lipo and OM-Lipo in 37 °C 1× PBS for 48 h, using coumarin-6 instead of carmustine, which is labile in aqueous solution [35]. The encapsulation efficiency of M-Lipo and OM-Lipo for carmustine and coumarin-6 was observed to be over 97% (Figure 4A). In addition, Briuglia et al. reported that in the phospholipid/cholesterol molar ratio (7:3), used in liposome synthesis, an increase in the cholesterol ratio reduces the encapsulation efficiency of hydrophobic drugs [36]. Therefore, in the present study, DSPC/cholesterol was used at a molar ratio of 7:3 for the release profile, and OM-Lipo were synthesized by additionally adding oleosin at 2% (*w*/*w*) of total lipids.

Consequently, in the early phase (<10 h) of the release profiles of M-Lipo and OM-Lipo, less than 30% of the release of the total loaded drug was observed in both (Figure 4B). A slightly higher drug release was observed from OM-Lipo before 12 h, but thereafter, M-Lipo showed higher release. Sequentially, more than 50% of the loaded carmustine was released in the M-Lipo group within 30 h, while less than 40% was released in the OM-Lipo group. After that, an improved sustained release of the drug was observed overall in OM-Lipo. These results suggest that liposomes synthesized via a microfluidic chip do not show a clear initial burst release of the loaded hydrophobic drug, and that the oleosin coating suppressed the release of the hydrophobic drug from the phospholipid bilayer.

### 3.5. In Vitro Cytotoxicity Studies

**Figure 5:**
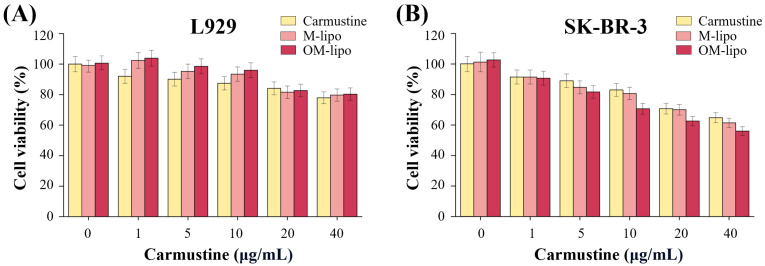
Cell viability of (**A**) normal cells (L929) and (**B**) cancer cells (SK-BR-3) according to treatment with carmustine (free drug), or the same drug concentration loaded onto M-Lipo and OM-Lipo.

A WST-1 assay was performed to investigate the cytotoxicity and cellular uptake rate of M-Lipo and OM-Lipo. First, the dose-dependent cytotoxicity of carmustine and the cytotoxic response to the fabricated carrier were investigated using mouse fibroblast L929 cells. In free carmustine treatment (positive control), cell viability decreased as the concentration increased (Figure 5A). Experiments using DDS showed that treatment with M-Lipo and OM-Lipo, loaded with the same concentration of free carmustine, showed lower cytotoxicity than the free drug treatment. In particular, the highest cell viability was observed with OM-Lipo treatment. These results show that the delivery of carmustine through LNPs has lower cytotoxicity compared to the free drug form. According to previous studies, reducing particle aggregation by surface modification can reduce the cytotoxicity of drug delivery through nanoparticle-based DDS [37]. However, additional experiments are needed to determine the reason for the highest cell viability observed with OM-Lipo treatment.

Then, to confirm that the LNPs increased the delivery efficiency, the effect of free drug, M-Lipo, and OM-Lipo carmustine delivery on the cancer cells’ viability (SK-BR-3) was evaluated. The experimental data clearly show that the cell viability of SK-BR-3 decreased with increasing carmustine concentration in all experimental groups (Figure 5B). In addition, while free carmustine did not show a significant change in anticancer effect against the cancer cell line when it was increased from 1 to 5 μg/mL, carmustine loaded into the carrier did. The difference in drug delivery efficiency between free drug and DDS treatment is attributed to drug carrier absorption via endocytosis [38]. The lowest cell viability, compared to the same drug concentration, was observed in OM-Lipo; at a carmustine concentration of 40 μg/mL, a cell death rate close to IC50 was observed only in OM-Lipo. DDS platforms based on LNPs can improve drug delivery efficiency by improving the half-life, dispersibility, and solubility of hydrophobic drugs [39]. In addition, because the oleosin coating can improve particle dispersion and membrane stability (Figure 2B), it is assumed that increasing the delivery efficiency of the drug was achieved with OM-Lipo. Therefore, the above results suggest that carmustine treatment through OM-Lipo can reduce its cytotoxicity and increase drug delivery efficiency to cancer cells.

## 4. Discussion

Most of the newly developed anticancer drugs have low solubility in water, so drug delivery using LNPs, which have high solubility for poorly soluble drugs, are considered a powerful tool in anticancer treatment [13]. In particular, small LNPs (<100 nm diameter) have been reported to have a passive targeting ability against solid tumors through the EPR effect [40]. Among the various types of LNPs, M-Lipo are synthesized through self-assembly on a microfluidic chip, enabling synthesis to the nano size (<100 nm) without strong physical/chemical reactions, such as high-press homogenization and harmful organic solvents [23,41]. In addition, their drug loading capacity was higher than that of liposomes prepared via traditional methods (i.e., film hydration) (Table 1) [42]. Furthermore, according to recent LNP surface modification studies, a membrane protein called oleosin was reported to improve the membrane stability of LNP, reduce aggregation, and affect the sustained release of drug [7]. Therefore, in this study, we discussed the preparation of OM-Lipo by adding oleosin in the microfluidic LNPs formulation process and their outstanding potential as anticancer drug carriers.

Normally, since LNP aggregation easily occurs in storage (especially in low temperature), DDS studies suggest the use of various surface modifications, including PEGylation, to prevent this aggregation [52]. Those techniques affect the physicochemical properties of the LNPs’ membrane and may impart various functionalities to them [25]. A study analyzing the emulsification properties of oleosin reported that its amphipathic structural characteristics can improve the integral physicochemical stability of the LNPs’ membrane [26,53]. As such, oleosin’s hydrophobic stem parts are reported to affect the binding between phospholipids’ hydrophobic tails in the phospholipid bi-layer, and their hydrophilic head part stabilizes the hydrophilic surface of LNPs [54]. Therefore, oleosin is considered to suppress aggregation between coated particles and improve the stability of emulsifiers [55,56]. The particle characterization and AFM-based surface imaging analysis performed in this study showed that a 2% (*w*/*w*) oleosin coating improved the dispersion of M-Lipo and suppressed particle aggregation during storage. The results presented indicate that the aforementioned oleosin coating may indeed have a significant effect on membrane stability in LNPs. However, it was confirmed that the use of >2% (*w*/*w*) oleosin coating significantly increased the particle size of OM-Lipo. As described by Guzha et al. (2023), this suggests that the addition of high concentrations of oleosin may negatively affect the stability of coated particles [54].

The burst release of drugs can lead to serious unintended side effects in the body, so drugs must be integrated into DDS that provide sustained drug release [57]. LNPs are considered excellent partners for anticancer drugs due to their high biocompatibility, and encapsulation efficiency for hydrophobic drugs, based on their lipophilic internal environment, but their initial drug burst release limits their potential applications [58]. Therefore, the use of surface modification based on various natural substances such as chitosan has been proposed to control excessive drug release from LNPs in the early stage of administration [59]. Oleosin has been reported to stabilize the phospholipid-base membrane and effectively increase the sustained drug release of LNPs [60]. Li et al. suggested the possibility that oleosin can slightly suppress the drug release of LNPs [28]. Based on Coumarin-6, in vitro release profiles were performed for M-Lipo and OM-Lipo, respectively. The sustained release of hydrophobic molecules from OM-Lipo was significantly improved compared to M-Lipo throughout the entire release phase. This is thought to be due to the stabilization of the phospholipid bilayer by the oleosin coating, resulting in the inhibition of hydrophobic molecule release from the membrane [26]. However, an explanation is needed for the slightly higher drug release from OM-Lipo that occurred before 6 h. The hydrophobic stems of oleosin and cholesterol are located in the phospholipid bilayer of OM-Lipo (Figure 1) [28]. Therefore, the above results can suggest that cholesterol and oleosin’s hydrophobic residues may compete with the hydrophobic drug for positions within the phospholipid bilayer, thereby increasing the amount of drug released from liposomes at the beginning of administration. However, to confirm the exact mechanism of this change in release tendency due to the oleosin coating, additional verification experiments are required.

Most recently developed anticancer drugs act based on specificity for therapeutic target classes that require more lipophilic compounds for affinity to targets, such as kinases and ion channels; therefore, most of them have strong hydrophobicity [61,62]. Among them, carmustine is a traditional hydrophobic anticancer drug that has an anticancer effect. However, its poor solubility and low specificity have led to toxicity to various non-target organs [63]. It has been reported that the delivery of anticancer drugs through highly biocompatible carriers such as LNPs can improve drug distribution in the blood circulation and reduce cytotoxicity [25,64]. In addition, the functionalization of LNPs through an oleosin coating is thought to improve drug delivery efficiency by affecting particle dispersion and drug release. Based on the L929-based cytotoxicity test results, OM-Lipo were able to reduce the toxicity of carmustine in vitro compared to the free drug and M-Lipo (Figure 5). In the drug delivery efficiency test for SK-BR-3, OM-Lipo showed a slightly higher delivery efficiency than the other two. Based on previous studies, these results are thought to be due to the improved dispersion and enhanced sustained release of LNPs by the oleosin coating [28,64]. However, further studies are needed to confirm the more exact mechanism of this phenomenon.

As a result, OM-Lipo have low cytotoxicity and better drug delivery efficiency than M-Lipo, showing the possibility of being used for delivering various substances, not only in the biomedical field but also in various fields such as food and cosmetics. However, research results on their immunogenicity, which cannot be ignored when using an oleosin coating, are still lacking [65]. Studies on food allergies have reported the possibility that oleosin may be included in the proteins that may cause allergic reactions to peanuts [66,67]. Therefore, in order to confirm the versatility of OM-Lipo, additional immunogenicity verification experiments for oleosin derived from various plants are required.

## 5. Conclusions

Recent strong collaborations between lipid-based DDS and surface modification have demonstrated the potential of lipid-nanoparticles in chemotherapy for cancer treatment. In the present study, we suggested a new method for synthesizing oleosin-coated M-Lipo (OM-Lipo) based on self-assembly and evaluated their capability as anticancer drug carriers. Consequently, the oleosin coating improved M-Lipo dispersibility and prevented particle aggregation for a long period of time under refrigerated storage conditions (4 °C). Moreover, OM-Lipo showed a more sustained release of the hydrophobic drug than M-Lipo, around body temperature. Drug delivery efficiency testing confirmed that OM-Lipo show low cytotoxicity against normal cells (L929) and better drug delivery efficiency of the anticancer drug to cancer cells (SK-BR-3) than the free drug and M-Lipo. These results suggest that the oleosin coating can expand the application range of M-Lipo and may be used as one of the best carriers for delivering various substances to the body. However, investigation into the exact mechanism by which an oleosin coating improves the membrane stability of M-Lipo, and the potential immunogenicity that can occur from using this surfactant protein under various routes of administration are still lacking. Therefore, additional experiments are needed to analyze the effect of the oleosin coating on a liposome’s membrane fluidity and verify its potential immunogenicity.

## Figures and Tables

**Table 1:** Comparative analysis between microfluidic chip-based or conventional liposome synthesis methods.

	Conventional Production Method		Microfluidic System	
	Thin-Film Hydration	Ethanol/Ether Injection	M-Lipo	OM-Lipo
Particle Size	>1000 nm	>200 nm	5~200 nm	>100 nm
For Nanoscale Synthesis	Sonication and Extrusion		Self-assembly	Self-assembly
Synthesis Speed	Slow	Normal	Fast	Fast
Encapsulation Efficiency	Low	Normal	High	High
Particle Size Distribution	Low Consistent	Low Consistent	Consistent	High Consistent
Particle Stability	Low	Low	Low	High
Quantity Production	Relatively Low	Normal	High	High
Organic Solvent Removal Method	Dry	Dialysis	Dialysis	Dialysis
Reference	[43,44,45]	[46,47,48]	[49,50,51]	This study

## Data Availability

The raw data supporting the conclusions of this article will be made available by the authors on request.

## Supplementary Materials

The following supporting information can be downloaded at: https://www.mdpi.com/article/10.3390/ma17225550/s1, Figure S1: Fabricated microfluidic device and design; Figure S2: Carbon Film Heater with Temperature Controller for DSPC Phase Transition; Figure S3: Instability analysis of carmustine in aqueous phase using RP-HPLC; Figure S4: SDS-PAGE gel image for showing the different concentration of Rapeseed oleosin extracted by MHE methods; Figure S5: Aggregation of DPSC inside a microfluidic channel; Figure S6: Atomic force microscope-based 3D image surface analysis of M-lipo and OM-lipo; Table S1: Information on materials used for SDS-PAGE analysis.
