# Supplementary material for: Microfluidic Fabrication of Oleosin-Coated Liposomes as Anticancer Drug Carriers with Enhanced Sustained Drug Release
Source: Materials (Basel). 2024 Nov 13;17(22):5550. doi: 10.3390/ma17225550 (PMC11595445; doi:10.3390/ma17225550)
Supplement: Supplementary file 1 [file materials-17-05550-s001.zip › materials-3256102-supplementary.pdf]

Supporting Information

Microfluidic Fabrication of Oleosome-Coated Liposomes as Anticancer Drug Carriers with Enhanced Sustained Drug Release

Yoseph Seo [1,†], Yeeun Woo [1,†], Byeolnim Oh [2], Daehyeon Yoo [1], Hyeok Ki Kwon [1], Chulhwan Park [1], Hyeon-Yeol Cho [3], Hyun Soo Kim [2,*], and Taek Lee [1,*]

[1] *Department of Chemical Engineering, Kwangwoon University, 20 Kwangwoon-Ro, Nowon-Gu, Seoul 01897, Republic of Korea*

[2] *Department of Electronic Engineering, Kwangwoon University, 20 Kwangwoon-Ro, Nowon-Gu, Seoul 01897, Republic of Korea*

[3] *Department of Bio & Fermentation Convergence Technology, Kookmin University, 77 Jeongneung-ro, Seongbuk-gu, Seoul 02707, Republic of Korea*

*Corresponding Authors' E-mail Ids: hyunsookim@kw.ac.kr (Prof. Hyun Soo Kim), tlee@kw.ac.kr (Prof. Taek Lee)

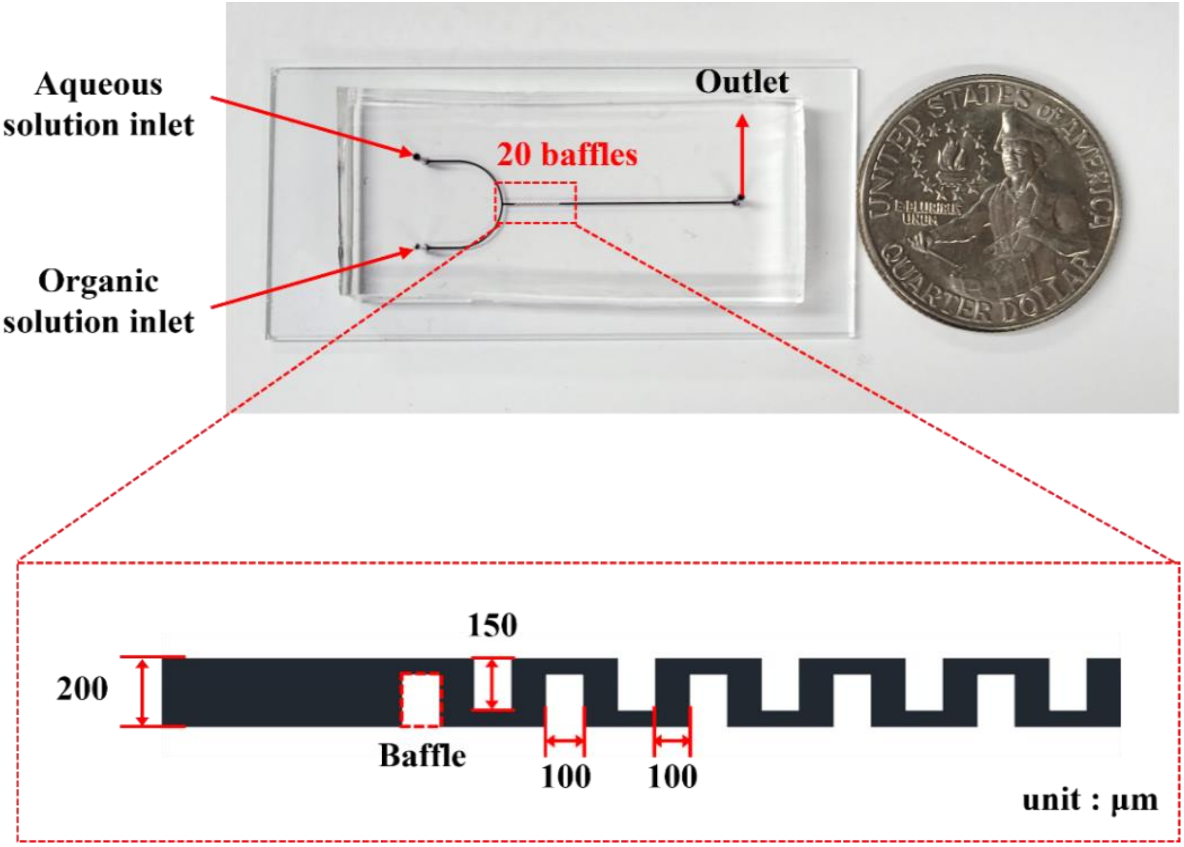

Figure S1. Fabricated microfluidic device and design. Twenty repeated baffles are arrayed within a 4 mm channel length for efficient fluid mixing and control.

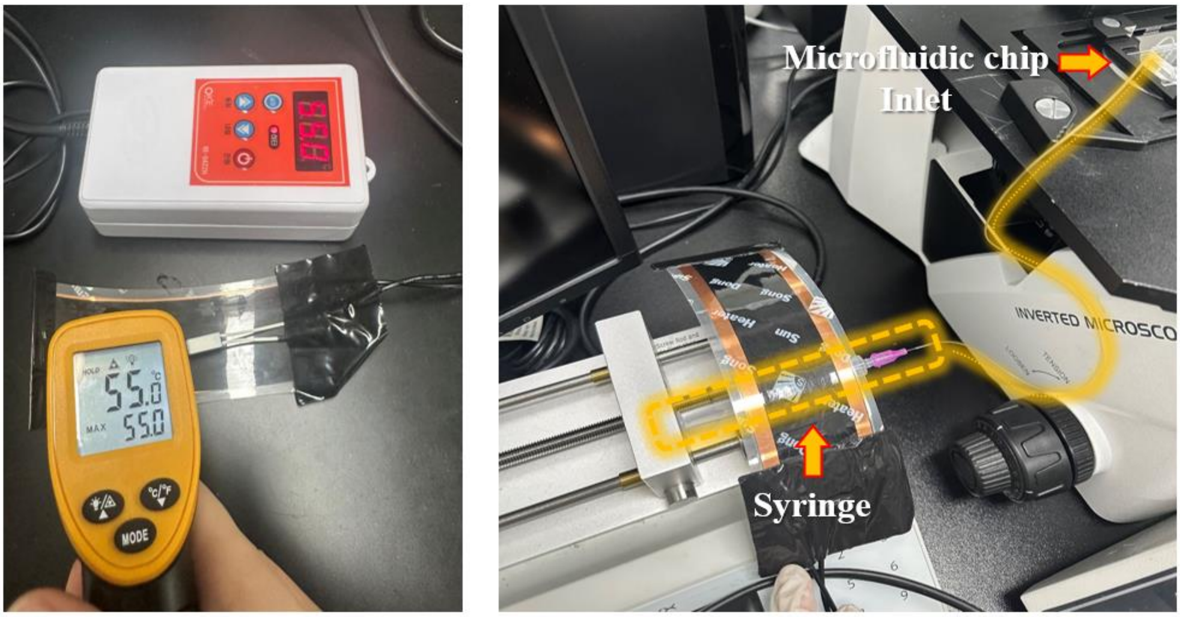

Figure S2. Carbon film heater and automatic temperature controller for a syringe heating to maintain the phase transition temperature of DPSC. Syringe and microbore tubing are marked with yellow color.

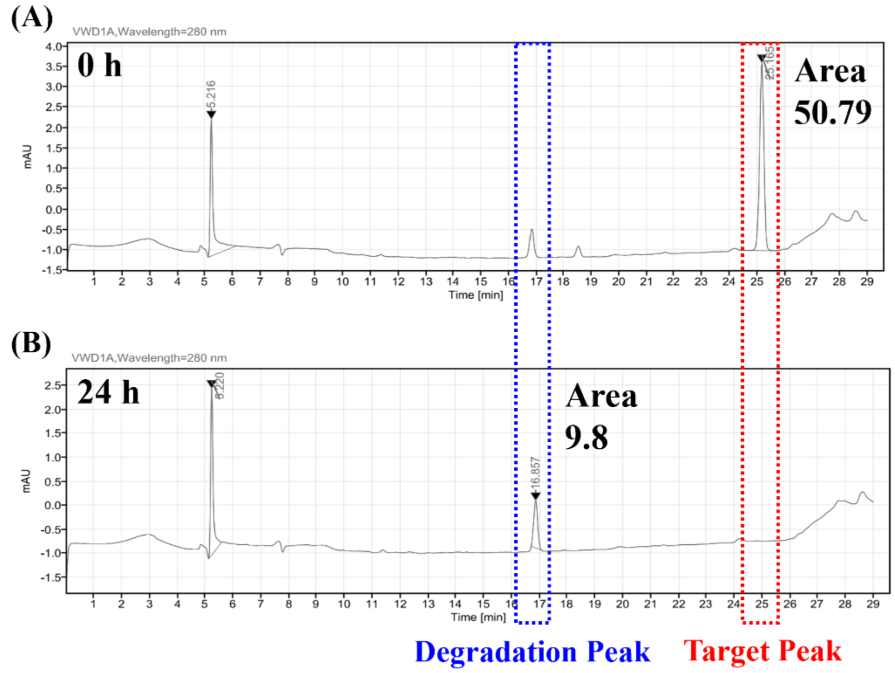

Figure S3. Testing the instability of carmustine in aqueous phase using RP-HPLC. 30 μg/mL carmustine at (A) 0 h and (B) 24 h after incubation in 37 °C 1X PBS.

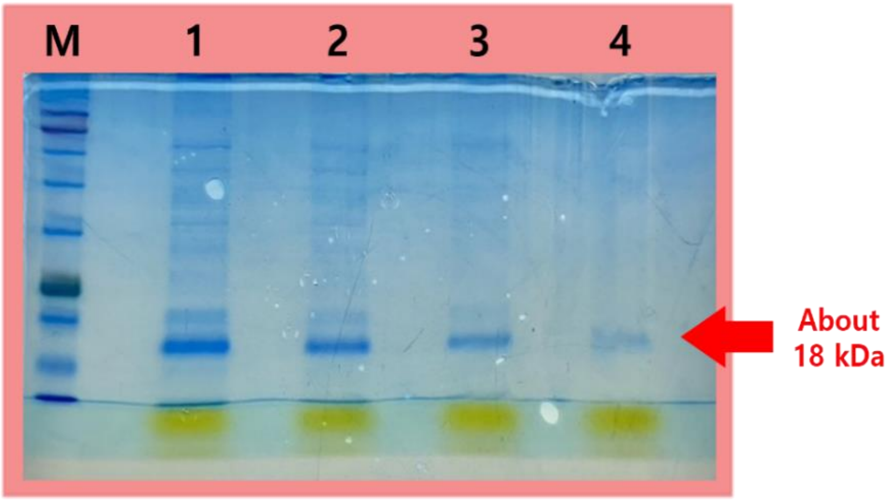

Figure S4. SDS-PAGE gel image for showing the different concentration of Rapeseed oleosin extracted by MHE methods. The gel shows the relationship between the band intensities of oleosin, and the concentrations of the protein loaded in the wells. The loaded concentration of oleosin in Lane 1 to 4 were 250 μg/mL, 125 μg/mL, 62.5 μg/mL, and 31.25 μg/mL. M is protein ladder marker.

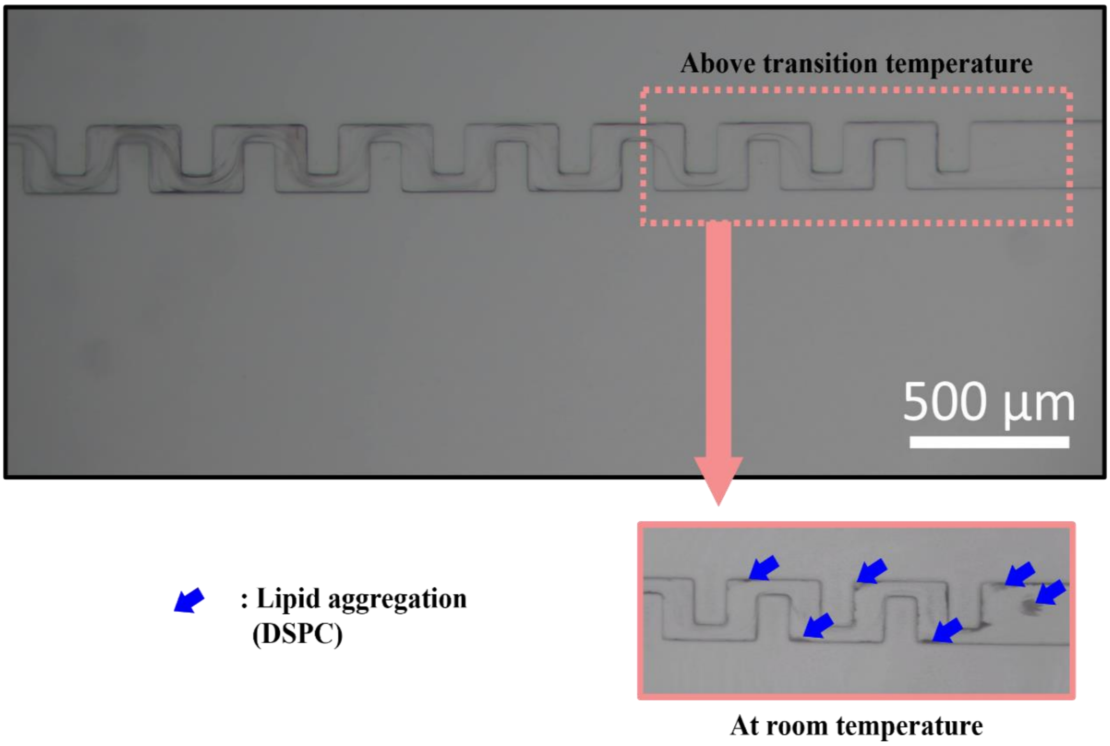

Figure S5. Aggregation of DPSC inside a microfluidic channel that occurs below the phase transition temperature of DSPC.

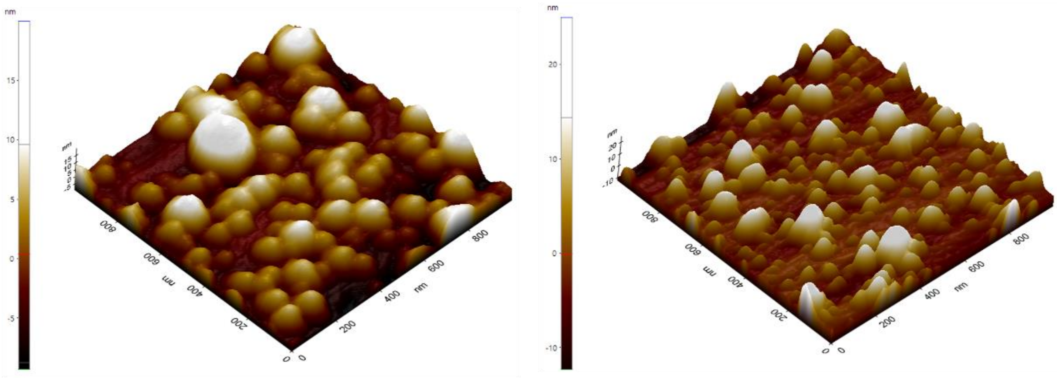

Figure S6. Atomic force microscope-based 3D image surface analysis of (A) M-Lipo and (B) OM-Lipo.

Table S1. Information on materials used for SDS-PAGE analysis.

	**Stacking Gel (Total: 1 mL)**	**Running Gel (Total: 5 mL)**
**H_2O**	0.688	1.65
**1M Tris-HCL (pH 6.8)**	0.125	1.25
**30% Acryl/bis-Acryl**	0.167	2
**10% SDS**	0.01	0.05
**10% APS**	0.01	0.05
**TEMED**	0.001	0.002

(Unit: mL)
